# Effects of Physicochemical and Biological Treatment on Structure, Functional and Prebiotic Properties of Dietary Fiber from Corn Straw

**DOI:** 10.3390/foods13131976

**Published:** 2024-06-22

**Authors:** Yijie Qin, Xinyao Fan, Ya Gao, Ping Wang, Juan Chang, Chaoqi Liu, Lijun Wang, Qingqiang Yin

**Affiliations:** College of Animal Science and Technology, Henan Agricultural University, Zhengzhou 450046, China; yijieqinhe@163.com (Y.Q.); fxy0115@126.com (X.F.); 15664016068@163.com (Y.G.); changjuan2000@126.com (J.C.); 15093389011@163.com (C.L.); wlj880626@163.com (L.W.); qqy1964@henau.edu.cn (Q.Y.)

**Keywords:** corn straw, dietary fiber, physicochemical treatment, biological treatment, functional property, prebiotic property

## Abstract

Corn straw is one kind of agricultural by-product containing 70–80% insoluble dietary fiber (IDF). In order to develop corn straw dietary fiber, this study was conducted to increase soluble dietary fiber (SDF) yield and improve the structure, functional and prebiotic properties of IDF and SDF from corn straw treated by alkali oxidation treatment, enzymatic hydrolysis, microbial fermentation and the combination of these methods. The results demonstrated that the yield of SDF was significantly increased from 2.64% to 17.15% after corn straw was treated by alkali oxidation treatment + *Aspergillus niger* fermentation + cellulase hydrolysis, compared with untreated corn straw. The SDF extracted from corn straw treated by alkali oxidation treatment + *Aspergillus niger* fermentation + cellulase hydrolysis (F-SDF) exhibited a honeycomb structure, low crystallinity (11.97%), good antioxidant capacity and high capacities of water holding, water solubility and cholesterol absorption and promoted short-chain fatty acids production by chicken cecal microbial fermentation in vitro. F-SDF enhanced the antibacterial activity against *Escherichia coli* and *Staphylococcus aureus* proliferations of *Lactobacillus plantarum* when it was used as a substrate for *Lactobacillus plantarum* fermentation. It could be concluded that the combined treatments could increase SDF yield from corn straw and improve its functional and prebiotic properties.

## 1. Introduction

Dietary fiber is one kind of functional ingredient with health-promoting and physiological functions for human and animals. It possesses water-binding and antioxidant capacity [1]. The physiological function and application of dietary fiber have been deeply studied in food and medicine, and it has sparked international interest owing to its crucial impact on animal health. Therefore, some researchers have started to develop novel dietary fiber from by-products abundant in fiber content [2,3,4].

Corn straw, as an agricultural by-product, primarily consists of cellulose and hemicellulose, which is an abundant and economical fiber resource. In China, approximately 240 million tons of corn straw are produced annually [5]. It could be an environmentally friendly and economical method to extract dietary fiber from corn straw. However, dietary fiber derived from corn straw has not been used in food and feed, since corn straw contains 70–80% insoluble dietary fiber (IDF) [6], and the soluble dietary fiber (SDF) content in corn straw is below 5% [7].

In general, SDF possesses a high-level fermenting ability and offers notable advantages in enhancing gastrointestinal health through regulating intestinal microbiota and promoting the growth of beneficial bacteria [8]. IDF can increase the volume of intestinal contents by absorbing water and promote intestinal peristalsis, thus promoting defecation and reducing the accumulation of toxins in the body [9]. Many beneficial effects attributed to dietary fiber are associated with SDF, which is popular due to its nutritional value as well as its superior bioactive and physicochemical properties [10]. The larger proportion of IDF can reduce the functional property, bioactivity and fermentation performance of dietary fiber food or feed [11]. The ratio of SDF and IDF in corn straw is seriously disproportionate, failing to meet the standard of high-quality dietary fiber with more than 10% SDF [12]. Therefore, the development of dietary fiber from corn straw must focus on increasing SDF content and decreasing IDF content for enhancing its functional properties.

Physical, chemical and biological modification methods have been widely used to improve the functional properties of fiber materials. Rice straw, buckwheat straw, maize straw core, tea residue and defatted rice bran were treated with alkaline hydrogen peroxide, sodium peroxide + high pressure, high temperature + enzyme hydrolysis and *Trichoderma viride* fermentation to obtain dietary fiber [2,7,12,13,14]. The bagasse fiber’s functionalities were improved by alkaline hydrogen peroxide + high temperature pressure modification [15]. Si et al. reported that *Aspergillus niger*-enzyme hydrolysis treatment was an effective and promising method to extract dietary fiber from Mesona chinensis Benth residues [16]. To date, there are few reports about the modification of corn straw dietary fiber.

In order to develop corn straw dietary fiber, the following experiments were conducted to (1) determine if the SDF yield from corn straw could be increased using a combination of physicochemical and biological methods including high pressure alkali oxidation, fungal fermentation and enzymolysis; (2) to examine the disparities in morphological structure and functional properties of SDF and IDF obtained from untreated and treated corn straw; and (3) to determine the prebiotic properties through in vitro fermentation. This study aimed to establish a more informed basis for making functional dietary fiber from corn straw for future applications.

## 2. Materials and Methods

### 2.1. Materials, Microbial Stains, Enzymes and Chemicals

The corn straw (maize cultivar Qiule 368) was collected from Zhecheng county (Shangqiu, China), crushed and passed through a 40-mesh sieve. *Aspergillus niger* (GDMCC 3.576) and *Lactobacillus plantarum* (ACCC10533) were purchased from Guangdong Microbial Culture Center (GDMCC, Guangzhou, China) and Agricultural Culture Collection of China (ACCC, Beijing, China), respectively. The pathogenic *Escherichia coli K88* (SHMCC D24607), *Staphylococcus aureus* (CVCC2257) and *Salmonella enteritidis* (CVCC3377) were purchased from Shanghai Microbiological Culture Collection (SHMCC, Shanghai, China) and National Center for Veterinary Culture Collection (CVCC, Beijing, China), respectively.

Thermostable α-amylase (CAS 9001-19-8), papain (CAS 9001-73-4), amyloglucosidase (CAS 9032-08-0), 1,1-diphenyl-2-picrylhydrazyl (DPPH, CAS 1898-66-4), O-phthalaldehyde (CAS 643-79-8) and resazurin sodium salt (CAS 62758-13-8) were purchased from Yuanye Biological Technology Co., Ltd. (Shanghai, China). The cellulase (347 filter paper unit (FPU)/g) was purchased from Xiasheng Industrial Group Co., Ltd. (Yinchuan, China).

### 2.2. Preparation of SDF and IDF from Corn Straw

The SDF and IDF were prepared according to the previous method [17] with minor modifications. The corn straw and 0.1 M phosphate-buffered saline (pH 7) were mixed at the ratio of 1:10 (*w*/*v*). Then, thermostable α-amylase (0.2 mL/g corn straw), papain (0.5%) and amyloglucosidase (0.5%) were added in sequence to efficiently hydrolyze the starch at pH 7, 95 °C for 30 min, protein at pH 7, 60 °C for 30 min and soluble sugar at pH 4.5 (pH was adjusted with 10 M hydrochloric acid), 60 °C for 30 min, respectively. To obtain SDF-enriched supernatant, the hydrolysate was filtered through a G3 crucible (pore size 16–30 μm) and rinsed thrice with 60 °C distilled water. The filtered residue was dried at 65 °C to obtain IDF. The supernatant was precipitated with 95% ethanol with 4 volumes at room temperature for 4 h and centrifuged for 10 min at 10,280× *g*. The sediment was dried at 65 °C to obtain SDF. TDF is the sum of SDF and IDF.

(1) $$\mathrm{SDF}\;\mathrm{yield}\;(\%)={100\times M}_{s}/M_{r}$$

(2) $$\mathrm{IDF}\;\mathrm{yield}\;(\%)={100\times M}_{i}/M_{r}$$

where *M_s_*, *M_i_* and *M_r_* are the dry weights of SDF, IDF and raw materials, respectively.

### 2.3. Corn Straw Treatments

#### 2.3.1. Alkali Oxidation Treatment

The straw was kept in 8% (*w*/*w*) sodium hydroxide with solid-to-liquid ratio of 1:2 (g/mL) and put in an autoclave sterilizer (LDZX-30 L, Shanghai Shenan Medical Instrument Factory, Shanghai, China) for 40 min at 121 °C and 0.1 MPa. Then, the reaction mixture was cooled to room temperature, and 6% (*v*/*v*) H_2_O_2_ (the volume of H_2_O_2_ was calculated based on the volume of liquid) was added to initiate a reaction for 4 h. The treated biomass was dried at 65 °C (without washing) and used for the following enzymatic hydrolysis and microbial fermentation.

#### 2.3.2. Enzymatic Hydrolysis

For enzymatic hydrolysis, 20 g corn straw, 300 FPU cellulase (15 FPU/g corn straw) and 100 mL distilled water were mixed in a flask. The flasks were shaken at 50 °C and 180 rpm to react for 2 h. The reaction was stopped by boiling for 30 min. The products were dried at 65 °C. The pH was adjusted to 4.5 with 10 M hydrochloric acid.

#### 2.3.3. Microbial Fermentation

*Aspergillus niger* was cultured on potato dextrose agar medium at 28 °C for 48 h. Subsequently, it was added to the spore suspension with a concentration of 1 × 10^7^ CFU/mL using distilled water. After, 20 g corn straw was mixed with 20 mL distilled water and sterilized, and then, 20 mL spore suspension of *Aspergillus niger* was added and mixed well. The microbial incubation was performed at 30 °C for 5 d. The products were dried at 65 °C. The pH of the fermentation system was adjusted to 5.5 with 10 M hydrochloric acid.

#### 2.3.4. Microbial Fermentation Plus Enzymatic Hydrolysis

During solid-state microbial fermentation, 300 FPU cellulase (15 FPU/g corn straw) was added. The performing protocol was the same as in Section 2.3.3.

### 2.4. Experimental Design

The designs of the optimal method for maximum SDF yield from corn straw were as follows:

Group 1: untreated corn straw.

Group 2: cellulase hydrolysis.

Group 3: *Aspergillus niger* fermentation.

Group 4: *Aspergillus niger* fermentation + cellulase hydrolysis.

Group 5: alkali oxidation treatment.

Group 6: alkali oxidation treatment + cellulase hydrolysis.

Group 7: alkali oxidation treatment + *Aspergillus niger* fermentation.

Group 8: alkali oxidation treatment + *Aspergillus niger* fermentation + cellulase hydrolysis.

The optimal method was selected for the next analysis.

### 2.5. Structural Characteristics

#### 2.5.1. Scanning Electron Microscope (SEM)

The SDF and IDF obtained from untreated and treated corn straw were coated with gold using an ion sputter coater (Cressington-108 Auto, Cressington Scientific Instruments Ltd., Watford, UK) and observed using FEI Q45 environmental scanning electron microscope (FEI Company, Hillsboro, OR, USA) at 3 kV with 5000× magnification. The SEM images were taken in secondary electron mode.

#### 2.5.2. X-ray Diffraction (XRD)

An X-ray diffractometer (D8 Focus, Bruker AXS GmbH, Karlsruhe, Germany) was used to analyze XRD patterns of SDF and IDF obtained from untreated and treated corn straw. The samples were scanned over the range of 2*θ* = 4–40° with a step size of 0.01°. The crystallinity index (CrI) was calculated according to the previous report [18].

(3) $$\mathrm{CrI}\;(\%)=100\times \left(I_{002}-I_{am}\right)/I_{002}$$

where *I*_002_ and *I_am_* are the diffraction intensity of the crystal structure at 2*θ* = 22.3° and the non-crystalline structure at 2*θ* = 18.1°, respectively.

#### 2.5.3. Fourier Transform Infrared (FT-IR) Spectroscopy

For the Fourier Transform Infrared (FT-IR) spectroscopy, 1 mg sample and 200 mg dried potassium bromide were thoroughly mixed, tableted and observed using a FT-IR spectrometer (Nicolet IS50, Thermo Fisher Scientific Inc., Waltham, MA, USA) with a spectral scan range of 4000–400 cm^−1^, 15 time scans and 4 cm^−1^ resolution.

### 2.6. Functional Property Analysis

#### 2.6.1. Water-Holding Capacity (WHC)

The WHC of SDF and IDF was determined using the previous method [19] with modification. For this, 1 g dietary fiber and 20 mL distilled water were mixed in a centrifuge tube. The mixtures were shaken at 180 rpm and 37 °C for 12 h and centrifuged at 10,280× *g* for 10 min to remove the supernatant. The wet sediment and centrifuge tube were weighed and dried at 105 °C.

(4) $$\mathrm{WHC}\;(g/g)=\left(M_{3}-M_{2}\right)/M_{1}$$

where *M*_1_, *M*_2_ and *M*_3_ are the weight of the dried dietary fiber, the dried sediment and centrifuge tube, and the wet sediment and centrifuge tube, respectively.

#### 2.6.2. Oil-Holding Capacity (OHC)

The OHC was measured using the previous method [2] with modification. Here, 1 g dietary fiber and 20 mL peanut oil were placed in a centrifuge tube and mixed well. The mixture was placed horizontally at 37 °C for 12 h, centrifuged at 10,280× *g* for 10 min. The sediment and centrifuge tube were weighed.

(5) $$\mathrm{OHC}\;(g/g)=\left(M_{3}-M_{2}\right)/M_{1}$$

where *M*_1_, *M*_2_ and *M*_3_ are the weight of dietary fiber, the dietary fiber and centrifuge tube, and the sediment and centrifuge tube, respectively.

#### 2.6.3. Water Solubility (WS)

The WS was determined according to the previous method [20] with modification. For this, 0.2 g dietary fiber and 4 mL distilled water were mixed in a centrifuge tube, kept at 37 °C for 12 h, and centrifuged at 10,280× *g* for 10 min. The supernatant was collected, dried and weighed.

(6) $$\mathrm{WS}\;(\%)={100\times M}_{1}/M$$

where *M*_1_ is the weight of supernatant after drying and *M* is the weight of sample.

#### 2.6.4. Cholesterol Absorption Capacity (CAC)

The CAC was determined using the method described by Xi et al. [17], where 10 mL fresh egg yolk and 90 mL distilled water were mixed to prepare diluted egg yolk emulsion. Then, 0.5 g dietary fiber sample and 10 mL diluted egg yolk emulsion were mixed and adjusted to pH 2 and 7. The mixture was shaken at 180 rpm and 37 °C for 12 h and centrifuged at 10,280× *g* for 10 min. The cholesterol in the supernatant was measured by the O-phthalaldehyde method [21].

(7) $$\mathrm{CAC}\;(\mathrm{mg}/g)=\left(M_{1}-M_{2}\right)/M$$

where *M*_1_ is the cholesterol contents of the yolk emulsion, *M*_2_ is the cholesterol contents after absorption and *M* is the weight of the sample.

#### 2.6.5. 1,1-Diphenyl-2-picrylhydrazyl (DPPH) Radical Scavenging Activity

The DPPH radical scavenging activity was determined using the previous method [22] with modifications, where 50 mg dietary fiber and 50 mL distilled water mixed to prepare the sample solution. Next, 2 mL dietary fiber solution was mixed with 2 mL 0.2 mM DPPH solution prepared by 95% ethanol and stored in the dark for 30 min at room temperature. Then, the absorbance was measured by spectrophotometer at 517 nm.

(8) $$\mathrm{DPPH}\;\mathrm{radical}\;\mathrm{scavenging}\;\mathrm{activity}\;(\%)=100\times \left[1-\left(A_{1}-A_{2}\right)/A_{0}\right]$$

where *A*_0_ is the absorbance of the control (2 mL distilled water and 2 mL DPPH), *A*_1_ is the absorbance of the experimental dietary fiber 1 (2 mL dietary fiber and 2 mL DPPH) and *A*_2_ is the absorbance of the experimental dietary fiber 2 (2 mL dietary fiber and 2 mL 95% ethanol).

### 2.7. Determination of Short-Chain Fatty Acids (SCFAs) by Cecal Microbial Fermentation In Vitro

#### 2.7.1. Preparation of Fermentation Inoculum

The intact ceca were taken from 20 42-day-old female Arbor Acres broilers, which was approved by the Animal Welfare and Ethical Committee of Henan Agricultural University (SKLAB-B-2010-003-01). The cecal contents were transferred into a sterile graduated bottle no later than 15 min postmortem and mixed with a 4-fold volume of sterile saline. Subsequently, the cecal mixture was filtered through four layers of sterile gauze and diluted with buffer [23] at a ratio of 1:9 to prepare the fermentation inoculum. In the entire process, the bottle was maintained at 38 °C in a water bath and continuously injected with CO_2_ to provide anaerobic conditions.

#### 2.7.2. Cecal Microbial Fermentation In Vitro

For the cecal microbial fermentation in vitro, 0.1% resazurin sodium salt solution was added to the fermentation inoculum at the ratio of 1:1, and CO_2_ was injected until the color of the fermentation inoculum changed from blue to colorless. Then, 30 mL fermentation inoculum and 0.3 g dietary fiber sample were accurately transferred into a 100 mL culture vial, followed by static cultivation at 38 °C for 24 h. Blanks with fermentation liquid but without substrate were used as the control.

#### 2.7.3. Determination of SCFAs

The SCFAs was determined using the previous method [24] with modifications. The fermentation liquid was centrifuged at 4 °C, 1644× *g* for 10 min, to obtain the supernatant. Then, 1 mL supernatant and 0.2 mL 20% (*v*/*v*) metaphosphoric acid were mixed and refrigerated for 30 min at 4 °C. Subsequently, the mixture was centrifuged at 10,280× *g* for 10 min. Next, 10 µL supernatant was diluted 100-fold and then filtered through a 0.22 µm filtration membrane. The SCFAs were measured by high-performance liquid chromatography (Agilent 1200, Agilent Technologies Inc., Santa Clara, CA, USA) with Sepax Carbomix H-NP (5%, 5 μm, 7.8 × 300 mm) (Sepax, Newark, NJ, USA).

### 2.8. Antibacterial Activity of Dietary Fiber and Lactobacillus plantarum Fermentation Liquid

*Lactobacillus plantarum* was cultured in Man Rogosa Sharpe (MRS) liquid medium at 37 °C for 24 h and diluted with distilled normal saline to achieve counts of 1 × 10^8^ CFU/mL. Then, 1 mL *Lactobacillus plantarum* solution was added into 100 mL sterilized MRS liquid medium containing 1% (*w*/*v*) dietary fiber, cultured at 37 °C for 48 h.

The antibacterial activity of fermentation liquid was determined using the Oxford cup method [25]. *Escherichia coli*, *Staphylococcus aureus* and *Salmonella enteritidis* were cultured in Luria–Bertani (LB) liquid medium at 37 °C for 24 h, and subsequently 50 μL pathogenic bacteria solution was evenly spread onto an LB solid plate. After complete absorption of the solution, the Oxford cup (8 mm diameter) was gently positioned in a vertical orientation, and 200 μL dietary fiber + *Lactobacillus plantarum* fermentation liquid was added to it. The plates were placed at 4 °C for 4 h and then incubated at 37 °C for 16 h. The diameter of the inhibition zone was recorded. The control group consisted of dietary fiber without *Lactobacillus plantarum* fermentation.

### 2.9. Statistical Analyses

The data were statistically analyzed using IBM SPSS 21.0 statistical software (SPSS Inc., Chicago, IL, USA) and expressed as mean ± standard deviation. The significance level at *p* < 0.05 was defined by Duncan’s test. Origin 2021 (Origin Lab Inc., Northampton, MA, USA) was used for graph construction.

## 3. Results and Discussion

### 3.1. Effect of Different Treatments on the Contents of SDF and IDF in Corn Straw

The contents of total dietary fiber (TDF), SDF and IDF in different treated corn straws are shown in Table 1. The content of SDF in corn straw treated by alkali oxidation + *Aspergillus niger* fermentation + cellulase hydrolysis (group 8) was the highest (17.15%), which increased by 6.5-fold compared to the untreated corn straw (2.64%). The content of IDF in the treated corn straw decreased to 43.59% from 76.24% in the untreated corn straw. Therefore, the percentage of SDF to IDF in the treated corn straw was 39.34%, which increased by 11.4-fold compared with that in untreated corn straw (3.46%), falling in the suggested well-balanced proportion range of SDF to IDF between 30% and 50% [26].

Compared with untreated corn straw (group 1), the contents of SDF and the ratio of SDF to IDF in other groups were significantly increased. Furthermore, the contents of SDF and the ratio of SDF to IDF in groups 2–4 were significantly lower than those in groups 5–8 combined with the alkali oxidation treatment, indicating the efficiency of alkali oxidation. This is because alkali oxidation treatment can break down lignin, hydrolyze hemicellulose, and expose cellulose [27] to make it easier for IDF to be converted to SDF [28]. The contents of TDF, SDF and IDF in the alkali oxidation + cellulase hydrolysis group (group 6) were significantly lower than those in group 5 with only the alkali oxidation treatment due to the more facile hydrolysis of IDF and SDF to be converted to monosaccharides by cellulase after corn straw is pretreated with alkali oxidation. The previous study reported that alkali oxidation (CaO + NaOH + H_2_O_2_) pretreatment combined with enzymatic hydrolysis increased the yield of reducing sugar to 322.42 mg/g from 11.45 mg/g [17], in agreement with this study. However, this result did not occur in group 8, due to the different pH and temperature between group 6 (pH 4.5, 50 °C) and group 8 (pH 5.5, 30 °C), which may affect cellulase’s capacity to convert cellulose into reducing sugar.

In addition, *Aspergillus niger* fermentation + cellulase hydrolysis in the untreated or alkali oxidation-treated corn straw (groups 4 and 8) could significantly increase the SDF content and the ratio of SDF to IDF, compared with only *Aspergillus niger* fermentation (groups 3 and 7), in agreement with a previous study [16]. The results indicate that the treatment of *Aspergillus niger* fermentation combined with cellulase hydrolysis could enhance IDF’s ability to be converted to SDF rather than monosaccharides. For further study, the alkali oxidation + *Aspergillus niger* fermentation + cellulase hydrolysis method was selected for comparison against the untreated corn straw.

### 3.2. Structural Characteristics

#### 3.2.1. SEM

**Figure 1:**
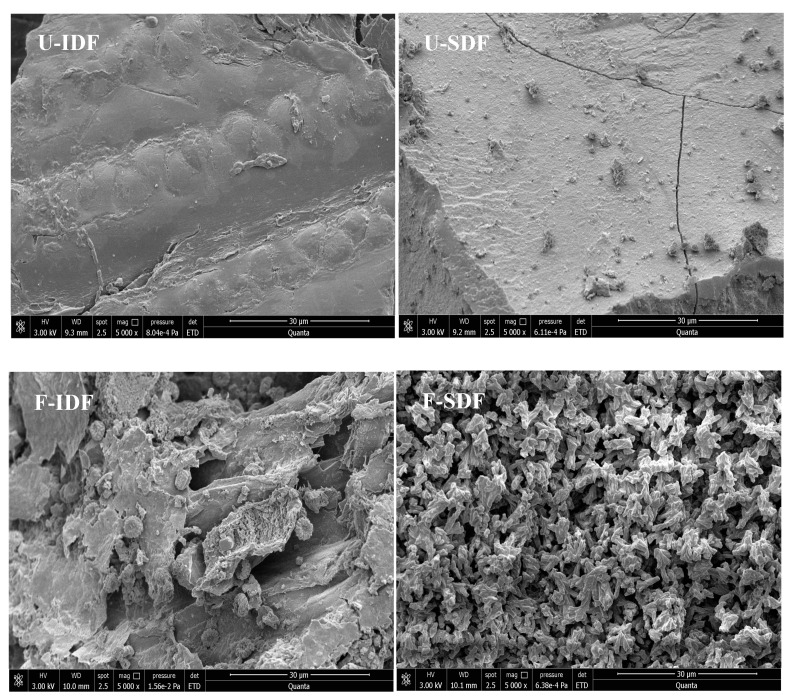
SEM images (5000×) of IDF and SDF with different treatments. Note: U-IDF: the IDF obtained from untreated corn straw; F-IDF: the IDF obtained from alkali oxidation treatment + *Aspergillus niger* fermentation + cellulase hydrolysis-treated corn straw; U-SDF: the SDF obtained from untreated corn straw; F-SDF: the SDF obtained from alkali oxidation treatment + *Aspergillus niger* fermentation + cellulase hydrolysis-treated corn straw.

As shown in Figure 1, the surfaces of IDF extracted from untreated corn straw (U-IDF) had a smooth and dense texture without any trace of erosion. In contrast, the surface morphology of IDF extracted from alkali oxidation treatment + *Aspergillus niger* fermentation + cellulase hydrolysis-treated corn straw (F-IDF) was further damaged after microbial fermentation and cellulase hydrolysis, resulting in fiber structure collapse accompanied by numerous grooves and cracks. Additionally, a lot of *Aspergillus niger* spores were adhered to the surface of F-IDF. The previous report showed that the alkaline oxidation + *T. koningii* fermentation + enzymatic hydrolysis-treated corn straw was friable and cellulose was broken down [27]. The surface of SDF obtained from untreated corn straw (U-SDF) was flat, with a smooth and dense texture without obvious pores, while the surface of SDF obtained from alkali oxidation treatment + *Aspergillus niger* fermentation + cellulase hydrolysis-treated corn straw (F-SDF) exhibited distinctive characteristics including irregular distribution, loose granularity and honeycomb structures, which increased the surface area and exposed the binding sites. The SEM results indicated that the treatments had significant effects on the microstructure of dietary fiber. The loose and porous structure of dietary fiber is beneficial to its functional properties, including enhanced WHC, OHC and CAC [2,20,29].

#### 3.2.2. XRD

**Figure 2:**
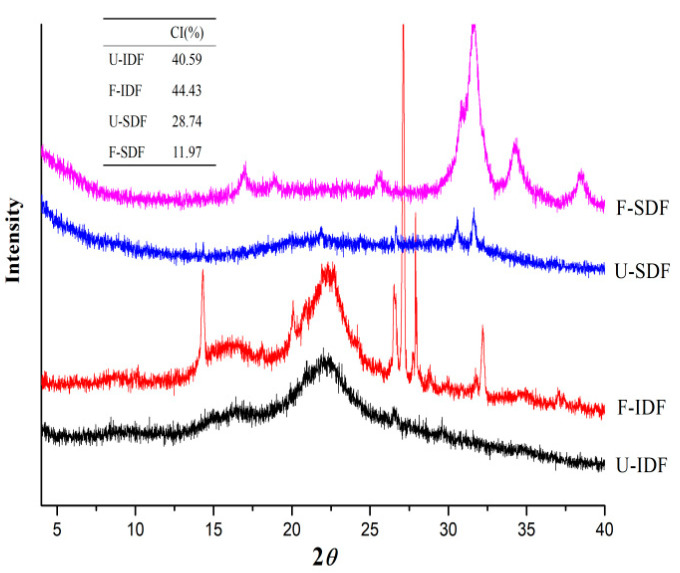
XRD of IDF and SDF with different treatments. Note: U-IDF: the IDF obtained from untreated corn straw; F-IDF: the IDF obtained from alkali oxidation treatment + *Aspergillus niger* fermentation + cellulase hydrolysis-treated corn straw; U-SDF: the SDF obtained from untreated corn straw; F-SDF: the SDF obtained from alkali oxidation treatment + *Aspergillus niger* fermentation + cellulase hydrolysis-treated corn straw.

As shown in Figure 2, IDF had distinctive characteristic diffraction peaks near 16.3° and 22.4°, representing the crystalline region of cellulose I type. The diffraction intensity and crystallinity of U-IDF and F-IDF were similar. The diffraction intensity and crystallinity of SDF were lower than those of IDF. After alkali oxidation treatment + *Aspergillus niger* fermentation + cellulase hydrolysis, the crystalline region of F-SDF disappeared and the crystallinity decreased to 11.97% from 28.74%, which indicates that the cellulose was decomposed and converted to small molecular saccharides. The crystallinity of SDF obtained from *Mesona chinensis* Benth residues treated by *Aspergillus niger* fermentation + enzyme hydrolysis reduced to 17.16% from over 30% in [16], which is almost the same as this study. The XRD results indicate that the treatment methods had remarkable effects on the crystalline region of fibers. The sharp diffraction peaks in F-IDF and F-SDF were NaCl crystals produced by adjusting the pH with HCl.

#### 3.2.3. FT-IR

**Figure 3:**
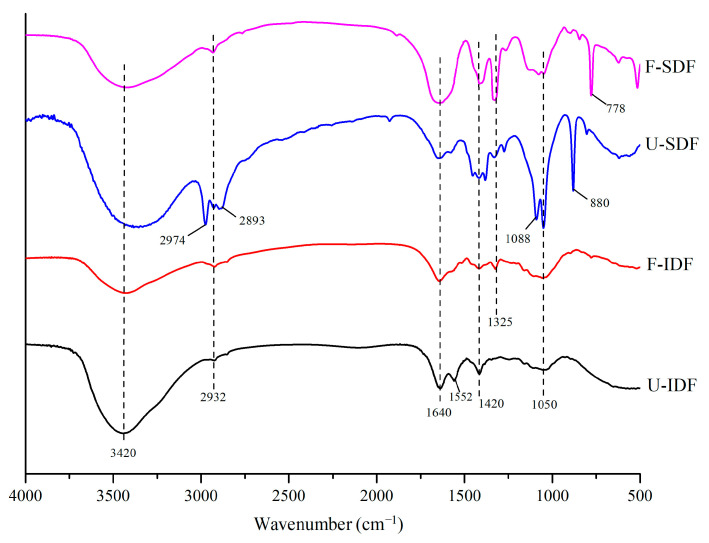
FTIR of IDF and SDF with different treatments. Note: U-IDF: the IDF obtained from untreated corn straw; F-IDF: the IDF obtained from alkali oxidation treatment + *Aspergillus niger* fermentation + cellulase hydrolysis-treated corn straw; U-SDF: the SDF obtained from untreated corn straw; F-SDF: the SDF obtained from alkali oxidation treatment + *Aspergillus niger* fermentation + cellulase hydrolysis-treated corn straw.

The peak near 3420 cm^−1^ was the stretching vibrations of O-H in the hydroxyl groups, and the peak of 2932 cm^−1^ was the stretching vibrations of C-H in the sugar methyl and methylene groups, which were the characteristic peaks of polysaccharides [30]. The absorption peak near 1640 cm^−1^ was the characteristic absorption of C=O for uronic acid [2]. As shown in Figure 3, the IDF and SDF samples showed absorption peaks near 3420 cm^−1^, 2932 cm^−1^ and 1640 cm^−1^. The peak strength was weaker in F-IDF during the range of 3420–3400 cm^−1^, compared with the untreated sample, indicating that the hemicellulose and cellulose contents in F-IDF were reduced. The F-SDF showed a weaker peak near 3420 cm^−1^ and 2932 cm^−1^, compared with U-SDF, suggesting that the hydroxyl, methyl and methylene groups decreased after treatments. However, the adsorption peak intensity at 1640 cm^−1^ in F-SDF was stronger than that in U-SDF, indicating that uronic acid increased after alkali oxidation + *Aspergillus niger* fermentation + cellulase hydrolysis. The bands at 1450 to 1200 cm^−1^ were the C-H bending vibrations of methylene and methyl groups in carbohydrates [28]. The peaks of F-SDF at 1420 and 1325 cm^−1^ were obviously stronger after alkali oxidation + *Aspergillus niger* fermentation + cellulase hydrolysis, suggesting that the structural carbohydrates were decomposed into smaller molecular carbohydrates. The vibration bands near 1088 cm^−1^ and 1050 cm^−1^ were C-O stretching vibrations in hemicellulose and cellulose [31], which were decreased in the F-SDF sample. The weak peak at 880 cm^−1^ observed in F-SDF implies that β-glycosidic linkages were destroyed compared with U-SDF [32]. However, the strong peak at 778 cm^−1^ in F-SDF was the stretching vibration of α-pyranose [20], indicating that alkali oxidation + *Aspergillus niger* fermentation + cellulase hydrolysis formed more contents of α-pyranose, mainly existing in SDF. In summary, the main functional groups of IDF and SDF were similar, but the strength of the functional groups was changed with different treatments, especially in the SDF sample.

### 3.3. Functional Characteristics

#### 3.3.1. WHC, OHC and WS

As shown in Table 2, the WHC sequences of IDF were F-IDF (3.77 g/g) < U-IDF (6.57 g/g). The results indicated that the alkali oxidation + *Aspergillus niger* fermentation + cellulase hydrolysis treatment reduced the WHC of IDF because of the decomposition of cellulose and hemicellulose [33]. After alkali oxidation treatment, the corn straw was fragile, the original mesh structures were destroyed, and parts of long chains of cellulose and hemicellulose were decomposed into short chains or monosaccharides with microbial fermentation and enzymatic hydrolysis [27]. This is the reason why the water binding capacity of IDF decreased after treatment. However, the WHC of SDF was F-SDF (11.64 g/g) > U-SDF (3.25 g/g). The highest WHC of F-SDF might be related to the loose granularity and honeycomb structures (Figure 1), which increased the surface area and exposed the binding sites [16,20,29]. In summary, the WHC sequences from high to low within the same treatment condition were F-SDF > F-IDF, U-IDF > U-SDF, indicating that the WHC of SDF was improved by treatment. High-WHC dietary fibers can enhance gut peristalsis to promote the excretion of toxins from body [34].

The OHC of IDF showed similar changing trends with WHC. The treatment increased the OHC of F-SDF but reduced the OHC of F-IDF. Under the same treatment, IDF exhibited a higher OHC than SDF. It has been previously reported that OHC decreases with decreasing cellulose and hemicellulose [33] and increases with the increasing porosity and more obvious honeycomb structure of dietary fiber [35]. The high OHC of dietary fiber can delay oil absorption in the body, which is beneficial to human health [16].

WS plays a crucial role in the development and application of dietary fiber. The high WS of dietary fiber leads to high-efficiency fermentation and porosity to make enzymes and bacteriophages spread inside the fiber [36]. The F-SDF exhibited higher WS than U-SDF, indicating that the alkali oxidation + *Aspergillus niger* fermentation + cellulase hydrolysis caused further degradation of IDF to be converted to soluble small molecule oligosaccharides.

The physicochemical characteristics of the materials indicate that SDF extracted from the treated corn straw has more advantages in WHC and WS, compared with SDF obtained from the untreated corn straw and IDF obtained from treated corn straw.

#### 3.3.2. CAC

Cholesterol is one of the culprits of cardiovascular disease. Dietary fiber as a therapeutic agent for hypercholesterolemia can combine with cholesterol to reduce its absorption [4]. As shown in Table 2, the CAC of IDF at pH 7 was higher than that at pH 2, indicating that IDF mainly absorbed cholesterol in the small intestine. At pH 7, the CAC of F-IDF was higher than that of U-IDF. F-SDF exhibited higher CAC at pH 2, while U-SDF exhibited higher CAC at pH 7. Liu et al. found that the CAC of SDF at pH 2 was higher than that at pH 7 [37], while other studies reported that the CAC of SDF at pH 7 was higher than that at pH 2 [2,38]. The SDF with different treatments showed a different CAC at pH 2 and pH 7, which might be related to the surface characteristics and structure. The CAC of SDF was higher than that of IDF regardless of using pH 2 or pH 7, which indicates that cholesterol adsorption mainly relied on SDF [39]. The evidence from this study suggested that the treatment of corn straw could effectively promote the cholesterol-combination capacity of SDF, which would have a positive effect on animal and human health.

#### 3.3.3. DPPH Radical Scavenging Activity

Scavenging of DPPH free radicals is one important indicator of antioxidant capacity. Table 2 showed that the DPPH radical scavenging capacities of F-IDF and F-SDF obtained by alkali oxidation + *Aspergillus niger* fermentation + cellulase hydrolysis were significantly higher than those of U-IDF and U-SDF. The reasons might be that *Aspergillus niger* fermentation + cellulase hydrolysis could improve the antioxidant activities of IDF and SDF by exposing a larger surface area and more functional groups [37], or that some active substances were produced by *Aspergillus niger* fermentation. Abdel-Wahhab et al. reported that the extracts of butylated hydroxytoluene, didodecyl 3,3′-thiodipropionate and pentasiloxane from *Aspergillus niger* cultured in potato dextrose broth (PDB) medium possess antioxidant activity [40]. However, the DPPH radical scavenging capacities of F-IDF were significantly higher than that of F-SDF, maybe due to those active substances removed by the extraction process of SDF.

### 3.4. Production of SCFAs from In Vitro Fermentation

SCFAs are the products of the symbiotic interaction between dietary fiber and gut microbiota, which played a crucial role in maintaining the integrity of the intestinal barrier, modulating immune responses, and preventing colorectal cancer and obesity [41]. The production of SCFAs from in vitro fermentation of IDF and SDF obtained from untreated and treated corn straw was evaluated, as shown in Table 3. The fermentation of U-IDF, F-IDF, U-SDF and F-SDF produced higher contents of total SCFAs than that of the blank group. The highest proportion of total SCFAs concerned acetic acid, followed by propionic acid, butyric acid, valeric acid, and isovaleric acid. Previous studies have demonstrated the inhibitory effects of acetic acid on tumor cells [42], as well as its potential in mitigating obesity and diabetes. Propionic acid can inhibit hepatic cholesterol synthesis [43]. In this study, the production of acetic acid, propionic acid and total SCFAs of F-IDF and F-SDF was significantly increased, compared with U-IDF and U-SDF, respectively, indicating that alkali oxidation + *Aspergillus niger* fermentation + cellulase hydrolysis treatment could significantly improve the fermentable properties of IDF and SDF and enhance SCFAs production. Previous research studies have reported that the treatment of dietary fiber could improve SCFA production from in vitro fermentation [44], which is consistent with this study. In addition, the ability of F-SDF to produce acetic acid, propionic acid and total SCFAs was the highest in all the samples, suggesting its potential prebiotic effects on the host’s health.

### 3.5. Effect of Dietary Fiber on the Antibacterial Activity of Lactobacillus plantarum

**Figure 4:**
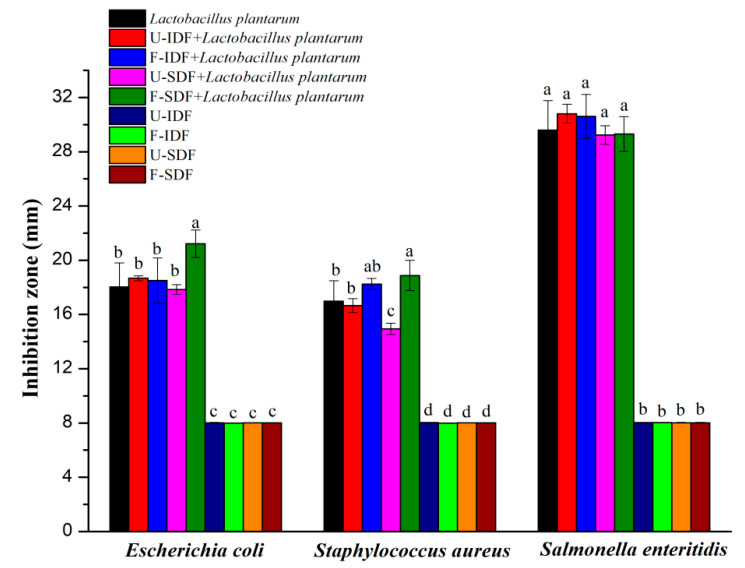
The effects of IDF and SDF with different treatments on the antibacterial activity (*n* = 5). Note: U-IDF: the IDF obtained from untreated corn straw; F-IDF: the IDF obtained from alkali oxidation treatment + *Aspergillus niger* fermentation + cellulase hydrolysis-treated corn straw; U-SDF: the SDF obtained from untreated corn straw; F-SDF: the SDF obtained from alkali oxidation treatment + *Aspergillus niger* fermentation + cellulase hydrolysis-treated corn straw. Different letters on each bar mean significant difference (*p* < 0.05).

*Lactobacillus plantarum* exhibits potent antibacterial activity against both *Escherichia coli* and *Staphylococcus aureus*, which benefits human and animal health [45]. The efficacy of dietary fiber derived from untreated and treated corn straw in enhancing the antibacterial effect of *Lactobacillus plantarum* is shown in Figure 4. Single U-IDF, F-IDF, U-SDF and F-SDF had no inhibitory effects on proliferations of *Escherichia coli*, *Staphylococcus aureus* and *Salmonella enteritidis*. A previous study reported that the SDF obtained from treated buckwheat bran showed better antibacterial effects [46], in disagreement with this study. A reason for this might be due to the different material and composition.

The bacteriostasis circle diameters of *Lactobacillus plantarum* fermentation liquid against *Escherichia coli* and *Staphylococcus aureus* proliferations were 18.03 mm and 16.97 mm, respectively. Compared to *Lactobacillus plantarum*, the F-SDF + *Lactobacillus plantarum* fermentation liquid significantly increased the antibacterial effects against proliferations of *Escherichia coli* (21.22 mm) and *Staphylococcus aureus* (18.88 mm). However, the U-SDF + *Lactobacillus plantarum* fermentation liquid exhibited insignificant impact against *Escherichia coli* proliferation (17.85 mm) and even demonstrated a significantly negative effect on *Staphylococcus aureus* proliferation (14.94 mm) when compared to single *Lactobacillus plantarum*. Both the U-IDF + *Lactobacillus plantarum* fermentation liquid and F-IDF + *Lactobacillus plantarum* fermentation liquid showed insignificant impact against proliferations of *Escherichia coli* and *Staphylococcus aureus* in comparison to single *Lactobacillus plantarum*.

Moreover, all dietary fiber samples + *Lactobacillus plantarum* fermentation liquid did not enhance antibacterial effects against *Salmonella enteritidis* proliferation in comparison to single *Lactobacillus plantarum*. The results demonstrate that alkali oxidation + *Aspergillus niger* fermentation + cellulase hydrolysis treatment improved the antibacterial activity of SDF + *Lactobacillus plantarum* fermentation against *Escherichia coli* and *Staphylococcus aureus* proliferations, which might be related to the high production of SCFAs [47].

## 4. Conclusions

This study indicated that SDF yield obtained from corn straw treated by alkali oxidation + *Aspergillus niger* fermentation + cellulase hydrolysis was 6.5-fold higher than that from untreated corn straw. F-SDF exhibited a honeycomb structure, low degree of crystallinity, high antioxidant capacity, and high capacities of WHC, WS and CAC in vitro. F-SDF exhibited the highest production of SCFAs by cecal microbial fermentation in vitro and enhanced the antibacterial activity of *Lactobacillus plantarum* against *Escherichia coli* and *Staphylococcus aureus* proliferations when it was used as a substrate for *Lactobacillus plantarum* fermentation. In summary, the SDF obtained from corn straw treated by alkali oxidation + *Aspergillus niger* fermentation + cellulase hydrolysis can be used as a functional ingredient in human food and animal diets. This study provides a novel strategy for dietary fiber extraction from corn straw and its potential application.

## Figures and Tables

**Table 1:** The IDF and SDF yield of corn straw treated with different methods (*n* = 3).

Groups	TDF (%)	IDF (%)	SDF (%)	SDF/IDF (%)
1	78.88 ± 0.19 ^b^	76.24 ± 0.33 ^a^	2.64 ± 0.52 ^e^	3.46 ± 0.83 ^e^
2	76.38 ± 1.31 ^c^	70.95 ± 1.60 ^b^	5.43 ± 0.34 ^d^	7.65 ± 0.65 ^d^
3	80.89 ± 1.70 ^a^	75.30 ± 1.83 ^a^	5.59 ± 0.30 ^d^	7.42 ± 0.52 ^d^
4	74.33 ± 0.21 ^d^	63.45 ± 0.95 ^c^	10.88 ± 0.74 ^b^	17.15 ± 1.42 ^c^
5	66.41 ± 0.56 ^e^	55.09 ± 0.33 ^d^	11.32 ± 0.40 ^b^	20.55 ± 0.72 ^b^
6	50.33 ± 0.46 ^g^	41.96 ± 0.69 ^e^	8.37 ± 0.42 ^c^	19.95 ± 1.26 ^b^
7	67.89 ± 1.12 ^e^	55.74 ± 0.39 ^d^	12.15 ± 1.44 ^b^	21.80 ± 0.94 ^b^
8	60.73 ± 0.82 ^f^	43.59 ± 1.00 ^e^	17.15 ± 0.86 ^a^	39.34 ± 0.57 ^a^

Note: group 1: untreated corn straw; group 2: cellulase hydrolysis; group 3: *Aspergillus niger* fermentation; group 4: *Aspergillus niger* fermentation + cellulase hydrolysis; group 5: alkali oxidation treatment; group 6: alkali oxidation treatment + cellulase hydrolysis; group 7: alkali oxidation treatment + *Aspergillus niger* fermentation; group 8: alkali oxidation treatment + *Aspergillus niger* fermentation + cellulase hydrolysis. Different letters in the same columns mean significant difference (*p* < 0.05).

**Table 2:** Functional characteristics of IDF and SDF (*n* = 3).

Items	U-IDF	F-IDF	U-SDF	F-SDF
WHC (g/g)	6.57 ± 0.26 ^b^	3.77 ± 0.19 ^c^	3.52 ± 0.16 ^c^	11.64 ± 0.24 ^a^
OHC (g/g)	5.41 ± 0.10 ^a^	3.68 ± 0.30 ^b^	2.69 ± 0.09 ^d^	2.97 ± 0.06 ^c^
WS (%)	6.08 ± 0.57 ^c^	3.08 ± 0.21 ^d^	40.16 ± 0.37 ^b^	92.69 ± 0.77 ^a^
CAC (mg/g) (pH 2)	14.82 ± 0.08 ^d^	19.66 ± 0.42 ^c^	50.13 ± 5.63 ^b^	198.81 ± 6.12 ^a^
CAC (mg/g) (pH 7)	29.66 ± 0.89 ^b^	88.45 ± 2.24 ^a^	85.24 ± 2.09 ^a^	88.93 ± 4.57 ^a^
DPPH (%)	35.94 ± 1.52 ^d^	73.58 ± 2.74 ^a^	42.34 ± 0.65 ^c^	58.99 ± 2.04 ^b^

Note: U-IDF: the IDF obtained from untreated corn straw; F-IDF: the IDF obtained from alkali oxidation treatment + *Aspergillus niger* fermentation + cellulase hydrolysis-treated corn straw; U-SDF: the SDF obtained from untreated corn straw; F-SDF: the SDF obtained from alkali oxidation treatment + *Aspergillus niger* fermentation + cellulase hydrolysis-treated corn straw. Different letters in the same rows mean significant difference (*p* < 0.05).

**Table 3:** Effects of SDF and IDF on production of short-chain fatty acids (SCFAs) from in vitro fermentation (*n* = 5).

Items	Blank	U-IDF	F-IDF	U-SDF	F-SDF
Acetic acid (mmol/L)	4.16 ± 0.28 ^e^	9.73 ± 0.43 ^d^	10.74 ± 0.40 ^c^	12.73 ± 0.47 ^b^	17.14 ± 0.14 ^a^
Propionic acid (mmol/L)	0.75 ± 0.12 ^e^	1.20 ± 0.15 ^d^	1.80 ± 0.08 ^c^	3.90 ± 0.13 ^b^	4.21 ± 0.07 ^a^
Butyric acid (mmol/L)	0.26 ± 0.04 ^d^	0.34 ± 0.08 ^c^	0.54 ± 0.05 ^b^	0.98 ± 0.05 ^a^	0.96 ± 0.02 ^a^
Valeric acid (mmol/L)	0.10 ± 0.01 ^c^	0.11 ± 0.01 ^bc^	0.12 ± 0.02 ^ab^	0.13 ± 0.01 ^a^	0.12 ± 0.01 ^ab^
Isovaleric acid (mmol/L)	0.08 ± 0.01 ^a^	0.06 ± 0.01 ^b^	0.06 ± 0.01 ^b^	nd	nd
Total SCAFs (mmol/L)	5.35 ± 0.31 ^e^	11.44 ± 0.43 ^d^	13.26 ± 0.55 ^c^	17.74 ± 0.36 ^b^	22.43 ± 0.23 ^a^

Note: U-IDF: the IDF obtained from untreated corn straw; F-IDF: the IDF obtained from alkali oxidation treatment + *Aspergillus niger* fermentation + cellulase hydrolysis-treated corn straw; U-SDF: the SDF obtained from untreated corn straw; F-SDF: the SDF obtained from alkali oxidation treatment + *Aspergillus niger* fermentation + cellulase hydrolysis-treated corn straw. nd: no detection. Different letters in the same rows mean significant difference (*p* < 0.05).

## Data Availability

The original contributions presented in the study are included in the article, further inquiries can be directed to the corresponding author.
